# Joint modeling and marker set selection significantly influence functional biomechanics in end-stage knee osteoarthritis: evidence from the sit-to-stand task

**DOI:** 10.3389/fbioe.2025.1677244

**Published:** 2025-10-13

**Authors:** Giovanni Spallone, Letizia Mancini, Arianna Carnevale, Stefano Campi, Emiliano Schena, Pieter D’Hooghe, Michael T. Hirschmann, Rocco Papalia, Umile Giuseppe Longo

**Affiliations:** ^1^ Fondazione Policlinico Universitario Campus Bio-Medico, Rome, Italy; ^2^ Research Unit of Measurement and Biomedical Instrumentation, Department of Engineering, Università Campus Bio-Medico di Roma, Rome, Italy; ^3^ Research Unit of Orthopaedic and Trauma Surgery, Department of Medicine and Surgery, Università Campus Bio-Medico di Roma, Rome, Italy; ^4^ Department of Orthopaedic Surgery and Sports medicine, Aspetar Hospital, Doha, Qatar; ^5^ University Clinic for Orthopedic Surgery and Traumatology, Kantonsspital Baselland, Bruderholz, Switzerland; ^6^ Department of Clinical Research, Research Group Michael T. Hirschmann, Regenerative Medicine & Biomechanics, University of Basel, Basel, Switzerland

**Keywords:** knee osteoarthritis, knee biomechanics, sit-to-stand, Istituto Ortopedico Rizzoli, calibration anatomical systems technique, joint modeling

## Abstract

**Introduction:**

The sit-to-stand (STS) movement represents a mechanically demanding task, particularly informative in patients with knee osteoarthritis. While three-dimensional optoelectronic motion capture is the gold standard for analyzing joint biomechanics, the influence of protocol selection remains poorly characterized in the context of STS. This study investigated protocol-induced variability in knee kinematics and kinetics by evaluating two widely used marker sets: the anatomical-based IOR and the cluster-based CAST, each combined with either inverse kinematics or a six degrees-of-freedom joint model.

**Materials and Methods:**

Twenty-four patients (mean age of 67 ± 5 years and BMI of 28.9 ± 3.8 kg/m^2^) with end-stage KOA (Kellgren-Lawrence grade 3 or 4) performed three STS trials, and biomechanical outputs were compared across the four resulting protocols using Mean Absolute Variability (MAV), Mean Absolute Differences (MAD), and Statistical Parametric Mapping (SPM).

**Results:**

Results revealed substantial variability across protocols, with the highest discrepancies observed in the sagittal plane: peak MAV reached 23.99° for knee flexion angle and 0.24 Nm/kg for knee flexion moment. Frontal and transverse parameters also showed clinically meaningful differences, particularly for knee adduction and internal rotation angles, with MAD values exceeding established thresholds. Differences were amplified when both markers set, and modeling strategy varied. In this context, cluster-based configurations showed reduced variability. SPM analyses revealed temporally localized differences, particularly at the initiation and final stabilization phases of the movement.

**Conclusion:**

These findings emphasize the critical role of protocol selection in motion analysis and its direct impact on the interpretation of knee biomechanics during functional tasks, highlighting the importance of adopting consistent and robust methodological frameworks to ensure clinical reliability and cross-study comparability.

**Clinical Trial Registration:**

https://clinicaltrials.gov/, identifier NCT06634654.

## 1 Introduction

Knee osteoarthritis (KOA) is a chronic joint disease affecting over 30% of adults aged over 65 years (Longo et al., 2024; Duffell L. et al., 2014; Liddle et al., 2013; Longo et al., 2015). It ranks among the most prevalent musculoskeletal disorders (Kour et al., 2021) and is currently one of the leading causes of disability worldwide (Zhang et al., 2021; Duffell et al., 2017; Longo et al., 2021). KOA is characterized by the progressive degeneration of articular cartilage, resulting in joint pain, stiffness, reduced mobility, and significant limitations in daily living activities (Zhang et al., 2020; Hussain et al., 2016; Lawrence et al., 1998; Longo et al., 2018).

Among these, the sit-to-stand (STS) movement is particularly relevant (Spyropoulos et al., 2013; Tarnita et al., 2020; O’Keeffe et al., 2023; Hughes et al., 1994; Higgs et al., 2019; Miura et al., 2018; Spallone et al., 2025), as it is performed multiple times daily and places high mechanical demands on the lower limbs (Sonoo et al., 2019; Petrella et al., 2021; O’Keeffe et al., 2023). This is particularly critical in older adults, who represent the primary population affected by KOA (Zhang et al., 2020; Pan et al., 2023; Longo et al., 2024). During STS, joint loading in the lower extremities is markedly higher than in many other functional tasks (Petrella et al., 2021), and patients with KOA often exhibit prolonged exercise duration due to quadriceps weakness (Patsika et al., 2011; Sonoo et al., 2019; Blessinger et al., 2025; Pan et al., 2024). This delayed execution increases the time under load for the knee joint, potentially exacerbating cartilage degeneration and functional decline (Sonoo et al., 2019).

Given its high sensitivity to musculoskeletal and neuromuscular deficits (Spyropoulos et al., 2013; Patsika et al., 2011; Petrella et al., 2021; Jeon et al., 2019), the STS task is routinely used in clinical rehabilitation and biomechanical assessment of knee joint function (Jeon et al., 2024). Quantitative analysis of joint kinematics and kinetics during this movement may support early identification of pathological patterns, objective monitoring of disease progression, and evaluation of functional recovery following conservative or surgical interventions.

In this context, several tools are currently available (Kour et al., 2021), including wearable inertial sensors (Van Lummel et al., 2013; Najafi et al., 2002; Janssen et al., 2005; Boonstra et al., 2006; O’Keeffe et al., 2023) and markerless motion analysis systems (Goffredo et al., 2009; Tanaka et al., 2019) for kinematic data, pressure and force platforms (Spyropoulos et al., 2013; Petrella et al., 2021; O’Keeffe et al., 2023; Anan et al., 2015) for kinetic insights, and surface electromyography (Petrella et al., 2021; Patsika et al., 2011; O’Keeffe et al., 2023; Miura et al., 2018) for muscles activity. When high spatial and temporal accuracy is required, three-dimensional optoelectronic motion capture (OMC) systems remain the gold standard (Cappozzo, 1984). These systems provide precise joint biomechanics, with minimal invasiveness and suitability for both medical and research environments (Robert-Lachaine et al., 2017b; Ferrari et al., 2010).

Nevertheless, the quality and clinical relevance of data derived from OMC systems depend not only on hardware specifications but also on the motion analysis protocol adopted (Ferrari et al., 2008; Duffell L. et al., 2014; Gorton et al., 2009; Mancini et al., 2025). A protocol encompasses a biomechanical model, marker set configuration, and standardized procedures for data acquisition, processing, and reporting (Ferrari et al., 2008; Andriacchi and Alexander, 2000; Gage, 1993; Sutherland, 2002; Sutherland, 2005), all of which significantly affect the interpretability of the output.

Among these, the marker set plays a pivotal role, as it directly defines segment coordinate systems and joint centers, influencing biomechanical variables computation (Ferrari et al., 2008; Mentiplay and Clark, 2018; Collins et al., 2009; Duffell L. et al., 2014; Żuk and Pezowicz, 2015; Buczek et al., 2010; Langley et al., 2021). Marker sets are generally classified as anatomical-based, where markers are placed on specific anatomical landmarks (Ferrari et al., 2008; Roy et al., 1991; Kadaba et al., 1990; Frigo et al., 1998; Leardini et al., 2007; Rabuffetti et al., 2019), or cluster-based, which use rigid marker plates attached to body segments (Ferrari et al., 2008; Benedetti et al., 1998; Duffell L. et al., 2014; Cappozzo et al., 1995). While the first provide more direct anatomical interpretation, they are susceptible to soft tissue artifacts and placement variability (Leardini et al., 2005; Della Croce et al., 2005; della Croce et al., 1999; Noonan et al., 2003). Cluster-based configurations, by contrast, offer greater tracking consistency during dynamic tasks but require careful calibration to associate the clusters with underlying anatomical structures (Duffell L. et al., 2014; Holden and Stanhope, 1998; Ji et al., 2023). Additionally, the biomechanical model defines joint degrees of freedom (DoF), segmental reference frames, and rotation conventions (Ferrari et al., 2008). Models vary considerably across protocols (Ferrari et al., 2008; Ji et al., 2023; Mentiplay and Clark, 2018; Pomarat et al., 2023; Fiorentino et al., 2020), and such differences can significantly influence the estimation of kinematic and kinetic parameters.

Despite its widespread use, motion analysis still lacks standardized guidelines, particularly for functional tasks like STS, which are highly sensitive to methodological variation. Although extensive literature has addressed the biomechanical outcomes related to this movement in the field KOA (Anan et al., 2015; Higgs et al., 2019; Miura et al., 2018; O’Keeffe et al., 2023; Pan et al., 2023; Patsika et al., 2011; Petrella et al., 2021; Sonoo et al., 2019; Spyropoulos et al., 2013; Tanaka et al., 2019; Tarnita et al., 2020), the influence of protocol selection remains currently underexplored, particularly in pathological populations such as end-stage KOA patients, as most previous analyses have focused on healthy individuals gait.

Our study aims to address this gap by evaluating the impact of two widely used marker set: the anatomical-based Istituto Ortopedico Rizzoli (IOR) (Leardini et al., 2007) and the cluster-based Calibration Anatomical Systems Technique (CAST) (Benedetti et al., 1998; Cappozzo et al., 1995). By comparing their outputs, we aim to quantify protocol-related differences and contribute to the standardization of motion analysis practices in the assessment of KOA.

## 2 Materials and methods

### 2.1 Participants

A total of twenty-four patients with end-stage KOA (i.e., Kellgren-Lawrence grade 3 or 4 (Kellgren and Lawrence, 1957)) scheduled for TKA were enrolled for this study at the Unit of Traumatology and Sports Medicine of Fondazione Policlinico Universitario Campus Bio-Medico. The study cohort included 24 participants (mean age of 67 ± 5 years, mean body mass index of 28.9 ± 3.8 kg/m^2^, 14 female 10 male). The study protocol was reviewed and approved by the Territorial Ethics Committee of Lazio Area 2 (Protocol Code: 179.24 CET2 cbm) and registered on ClinicalTrials.gov (ID: NCT06634654). Prior to participation, written informed consent was obtained from all subjects, and all experimental procedures were conducted at the Motion Analysis Laboratory of the of Fondazione Policlinico Universitario Campus Bio-Medico.

The inclusion criteria were symptomatic, end-stage KOA, aged 18 years or older, with functionally intact ligaments. Conversely, the exclusion criteria included neurological or other conditions affecting patients’ ability to join walking trials, inflammatory or infectious arthritis, previous articular fracture or knee surgery (excluding knee arthroscopy and meniscal surgery), and active tumors or pregnancy. Furthermore, individuals with evident joint deformities in the hip, knee, or ankle were excluded from the study, as such anatomical alterations impaired the reliable identification of bony landmarks necessary for accurate marker placement.

### 2.2 Data acquisition and processing

**FIGURE 1:**
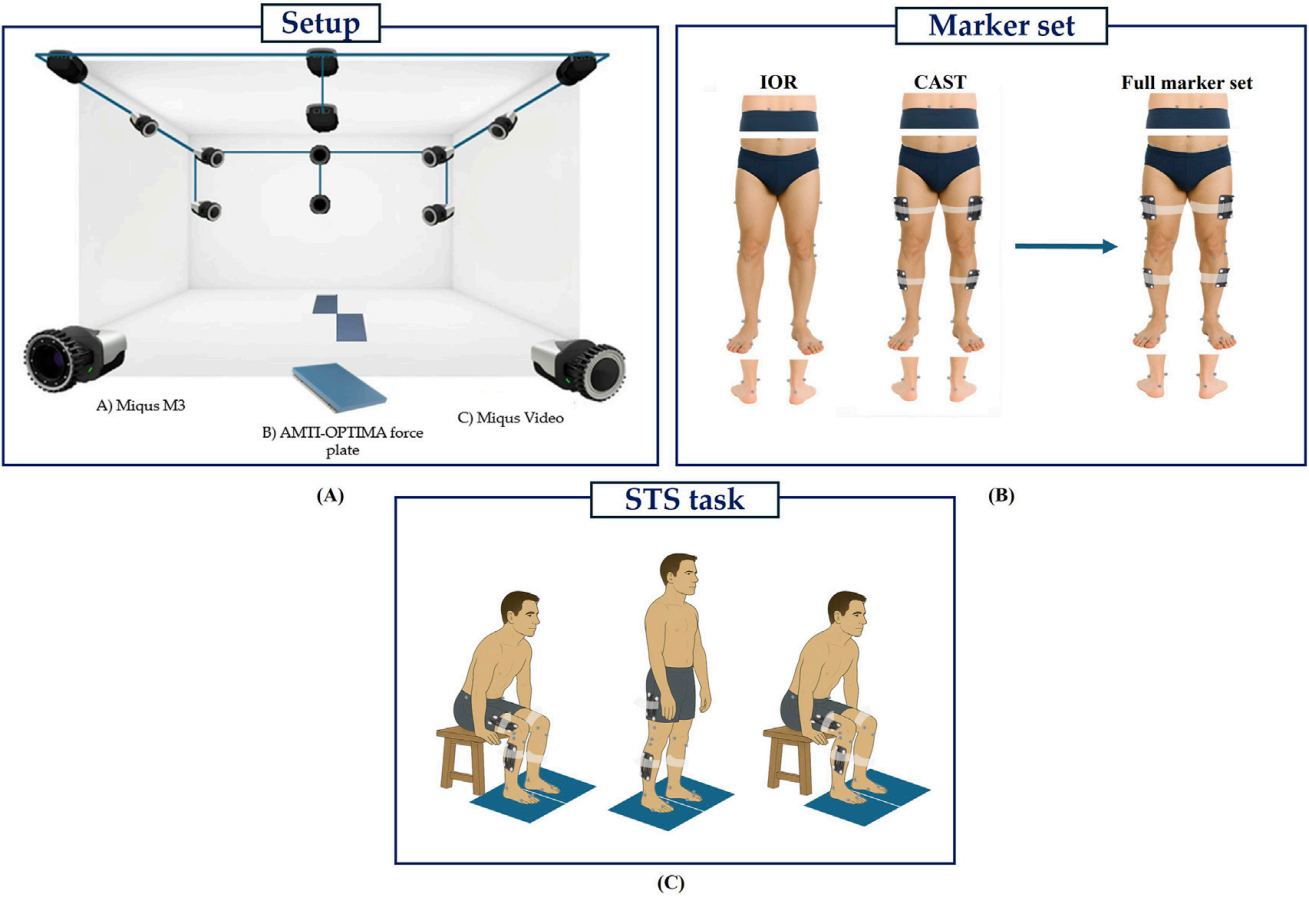
Experimental setup **(A)**; IOR, CAST and full marker set **(B)**, STS Task movement **(C)**.

Three-dimensional motion analysis was performed using a Qualisys™ stereophotogrammetric system (Qualisys AB, Gothenburg, Sweden) composed of ten infrared cameras (sampling frequency (F_s_) = 100 Hz) and two video cameras (F_s_ = 25 Hz) to support visual inspection and facilitate post-processing. Ground reaction forces (GRFs) were acquired through two embedded OPTIMA™ (Advanced Mechanical Technology Inc., Watertown, MA, United States) force platforms (F_s_ = 1000 Hz) fully synchronized with the OMC system. An overview of the experimental setup is summarized in Figure 1A.

To enable a direct comparison between protocols, both marker sets (IOR (Leardini et al., 2007) and CAST (Benedetti et al., 1998; Cappozzo et al., 1995)) were simultaneously applied to each participant. All markers were positioned by the same trained operator to ensure consistency and minimize inter-operator variability. The IOR (Leardini et al., 2007) marker set (Figure 1B) includes 26 anatomical markers (diameter: 12.5 mm) placed for static trial, and 20 for dynamic tasks. Markers were positioned bilaterally on the anterior and posterior superior iliac spines, lateral aspect of the greater trochanter, lateral and medial femoral epicondyles, proximal head of the fibula, tibial tuberosity, lateral and medial malleoli, insertion of the Achilles tendon on the calcaneus, and the dorsal margin of the first, second, and fifth metatarsal heads. On the other hand, the CAST (Benedetti et al., 1998; Cappozzo et al., 1995) marker set (Figure 1B) consists of 36 markers for static trial and 28 for dynamic tasks. This configuration integrates both anatomical markers and rigid clusters. Each cluster comprises a 131 × 80 mm rigid plate equipped with four retroreflective markers (diameter: 12.5 mm) mounted on brass inserts and is secured to the limb using a Fabrifoam SuperWrap strap to ensure stability during movement. The anatomical markers were positioned bilaterally on the anterior and posterior superior iliac spines, lateral and medial femoral epicondyles, lateral and medial malleoli, insertion of the Achilles tendon on the calcaneus, and the dorsal surface of the first, second, and fifth metatarsal heads. While four-marker clusters were securely attached to the lateral mid-thigh and mid-shank regions of both limbs. A complete representation of the full marker set configuration is shown in Figure 1B.

All participants first underwent a static acquisition in a T-pose (Robert-Lachaine et al., 2017a), which was used as a calibration trial for the definition of both the IOR and CAST biomechanical models. Subsequently, each participant performed three repetitions of the STS task (Figure 1C). The movement was initiated from a standardized chair with a fixed seat height of 43 cm (Higgs et al., 2019; Miura et al., 2018; Spyropoulos et al., 2013), with both feet placed entirely on the force platforms to ensure accurate recording of GRFs. The only constraint imposed was the initial posture, which required a knee flexion angle of 90° to standardize the starting condition across trials. Each repetition consisted of a full STS cycle: participants were instructed to rise from the chair, maintain an upright standing position for 2 seconds, and then return to the seated position. They performed the task at a self-selected speed and were allowed to use their upper limbs for support during both the ascent and descent phases. Particularly, arm assistance was intentionally permitted and applied in a standardized manner across all participants. During the ascent phase, patients used their hands to push off from the seat to initiate standing, while during descent, they placed their hands on the seat to control the return to a seated position. All trials were performed barefoot to minimize the risk of footwear-induced artifacts (Langley et al., 2021; Paquette et al., 2013).

Marker trajectories were processed in Qualisys Track Manager software (version 2024.2), including labelling, gap-filling, and filtering (fourth-order low-pass Butterworth filter with a cutoff frequency of 6 Hz (Zhang et al., 2021)). In addition, GRF data were filtered using a fourth-order low-pass Butterworth filter with a cutoff frequency of 50 Hz (Hewett et al., 2005) and all data streams were automatically synchronized via hardware triggering within the Qualisys acquisition system. Temporal alignment between kinematic and kinetic data was preserved during data import and model construction in Visual3D.

Four distinct biomechanical models were created in Visual3D™ (C-Motion, Inc., Germantown, MD, United States of America) by combining the two marker sets with two joint constraint methods: inverse kinematics (IK) and 6DoF (Kainz et al., 2016; Mentiplay and Clark, 2018; Mantovani and Lamontagne, 2017; Lu and O'Connor, 1999). The IK-based models constrained joint motion to three rotational DoF (Mentiplay and Clark, 2018), while the 6DoF models allowed both rotational and translational motion at the knee joint, providing an unconstrained segment tracking. The dual-modeling strategy was adopted to assess how different joint constraint assumptions may influence the estimation of clinically relevant biomechanical parameters in end-stage KOA. This approach resulted in four models: IOR_IK, IOR_6DoF, CAST_IK, and CAST_6DoF.

Joint centers were estimated using marker-based regression equations embedded in Visual3D. In particular, the hip joint center was computed based on the equations proposed by Bell et al., 1989, while the knee and ankle joint centers were defined as the midpoints between the lateral and medial epicondyles and malleoli, respectively. Segment coordinate systems, which served as reference frames, were defined based on well-established conventions reported in the literature (Benedetti et al., 1998; Cappozzo et al., 1995; Leardini et al., 2007), while inertial properties were estimated as fixed percentages of total body mass based on the default anthropometric equations implemented in Visual3D (Moreira et al., 2021). Knee kinematics were derived from three-dimensional joint angles using a Cardan rotation sequence with an X–Y–Z order (Williams et al., 2020), corresponding to flexion/extension (X), abduction/adduction (Y), and internal/external rotation (Z). While joint kinetics were computed through inverse dynamics (Marriott et al., 2019; Petrella et al., 2021; Selistre et al., 2017) and expressed as net external moments normalized to body weight to account for inter-subject variability.

### 2.3 Data analysis

Data were subsequently exported in MATLAB^®^ (MathWorks, R2023a) for further analysis. The STS cycle was segmented based on a combination of kinematic and kinetic criteria. The onset of movement was identified as the first frame in which the angular velocity of the hip joint crossed zero from negative to positive values, indicating the initiation of trunk flexion (Pan et al., 2023; Anan et al., 2015). The first buttocks-off event was detected when the vertical ground reaction force surpassed a minimal force threshold of 10 N, corresponding to the moment of seat lift-off (Sonoo et al., 2019; Anan et al., 2015). The standing phase was defined as the interval during which the hip joint center reached and maintained its maximum vertical position within a predefined spatial tolerance of ±3 cm, representing a stable upright posture (Spallone et al., 2025). Then, the second buttocks-off corresponded at the frame in which the vertical GRF dropped below 10N (Sonoo et al., 2019; Anan et al., 2015). Finally, the end of the movement was defined as the first frame after the descent during which the angular velocity of the hip joint crossed zero again, indicating a return to the initial position (Anan et al., 2015; Pan et al., 2023). All signals were temporally aligned through hardware synchronization within the acquisition system.

All variables were time-normalized over the full duration of the STS task to enable consistent comparisons across participants and trials. For each participant and protocol configuration, the three repetitions were subsequently averaged, and the standard deviation was obtained.

The Mean Absolute Variability (MAV) was computed across the four protocol configurations (IOR_IK, IOR_6DoF, CAST_IK, CAST_6DoF) for each variable of interest. Specifically, MAV was defined as the average of the absolute difference between the maximum and minimum values at each time-normalized frame, calculated over all protocol outputs (Ferrari et al., 2008; Leardini et al., 2007; Noonan et al., 2003; Gorton et al., 2009). This metric captured the total dispersion in kinematic and kinetic outputs, offering a macroscopic view of protocol-induced variability over the STS task.

To quantify the average discrepancy between models, Mean Absolute Differences (MAD) were computed for all pairwise protocol combinations. This metric was chosen for its robustness and interpretability in a clinical context. Notably, the absolute difference, rather than the signed difference (Collins et al., 2009), was chosen to ensure that subject-specific differences in opposite directions did not offset each other, thus preserving the true magnitude of inter-protocol variability (Langley et al., 2021). Importantly, regarding joint angles, the MAD was evaluated against a clinical threshold of 5°, with differences exceeding this value considered clinically meaningful (Langley et al., 2021; Nester et al., 2007).

Statistical Parametric Mapping (SPM) was used to analyse time-continuous joint biomechanics (Honert and Pataky, 2021; Park and Yoon, 2021; Beerse et al., 2024; Li et al., 2016) across the entire STS cycle. Unlike conventional approaches based on discrete variables (such as peak angles or range of motion), which may introduce regional bias and fail to account for inter-component covariance (Pataky et al., 2013; Beerse et al., 2024; Sole et al., 2017), SPM evaluates the full waveform, enabling the detection of statistically significant differences at specific time points within movement (Honert and Pataky, 2021; Park and Yoon, 2021; Beerse et al., 2024). This approach provides a more comprehensive and unbiased assessment of biomechanical patterns, particularly when differences are subtle or temporally localized (Honert and Pataky, 2021; Park and Yoon, 2021; Beerse et al., 2024). SPM was implemented in MATLAB^®^ using the open-source toolbox *spm1d* (v0.4, www.spm1d.org) (Park and Yoon, 2021; Honert and Pataky, 2021; Eerdekens et al., 2020). For each variable, pairwise comparisons between protocol configurations were performed using one-dimensional paired t-tests, and a nominal significance level of α = 0.05 was adopted (Park and Yoon, 2021; Honert and Pataky, 2021). SPM results were plotted and examined to identify temporally localized regions of statistically significant differences between protocols.

All analyses were performed bilaterally, including the side scheduled for TKA and its contralateral counterpart, to fully capture protocol-induced differences in biomechanical patterns.

## 3 Results

### 3.1 Protocol-induced variability: global assessment across the STS task

The MAV across the four protocol configurations revealed consistent inter-protocol discrepancies for both kinematic and kinetic parameters. Table 1 summarizes the MAV values computed over the time-normalized STS task for the surgical and contralateral sides. Among all parameters, the highest variability was observed in the sagittal plane, for both joint angles and moments. The KFA showed the greatest dispersion across protocols, with a MAV of 21.15° on the TKA side and 23.99° on the contralateral side. Similarly, the KFM exhibited the largest variability among kinetic measures, reaching 0.17 Nm/kg and 0.24 Nm/kg, respectively. Additionally, it is noteworthy that the contralateral limb consistently exhibited higher MAV values than the one scheduled for surgery.

**TABLE 1:** MAV values across all protocols for the limb scheduled for TKA (MAV_TKA) and the contralateral limb (MAV_contralateral).

Scheduled for TKA side	Unit	MAV_TKA	MAV_contralateral
KFA	[°]	21.15	23.99
KAA	[°]	14.86	15.61
KIRA	[°]	13.11	15.98
KFM	[Nm/kg]	0.17	0.24
KAM	[Nm/kg]	0.11	0.14
KIRM	[Nm/kg]	0.04	0.05

### 3.2 Pairwise agreement: protocol-by-protocol comparison

Table 2 reports MAD values for all knee joint angles and moments for both TKA and contralateral limbs.

**TABLE 2:** MAD for all knee joint angles and moments, computed for each pairwise protocol comparison. Results are reported separately for the limb scheduled for TKA and the contralateral limb. Kinematic values exceeding the clinical threshold of 5° are followed by*.

Protocols pair	KFA [°]		KFM [Nm/kg]	
	TKA	Contralateral	TKA	Contralateral
IOR_IK vs. IOR_6DoF	14.48*	17.39*	0.10	0.16
IOR_IK vs. CAST_IK	8.86*	10.65*	0.07	0.11
IOR_IKvsCAST_6DoF	14.87*	17.42*	0.11	0.16
IOR_6DoFvsCAST_IK	16.01*	16.95*	0.12	0.17
IOR_6DoFvsCAST_6DoF	5.02*	4.15	0.05	0.05
CAST_IKvsCAST_6DoF	15.75*	16.38*	0.12	0.15

	KAA [°]		KAM [Nm/kg]	
IOR_IK vs. IOR_6DoF	8.51*	8.92*	0.07	0.09
IOR_IK vs. CAST_IK	6.65*	7.52*	0.04	0.05
IOR_IKvsCAST_6DoF	5.73*	6.64*	0.05	0.05
IOR_6DoFvsCAST_IK	11.43*	11.84*	0.09	0.10
IOR_6DoFvsCAST_6DoF	9.97*	9.98*	0.07	0.09
CAST_IKvsCAST_6DoF	6.08*	6.39*	0.04	0.04

	KIRA [°]		KIRM [Nm/kg]	
IOR_IK vs. IOR_6DoF	9.70*	11.78*	0.02	0.04
IOR_IK vs. CAST_IK	6.31*	15.48*	0.01	0.02
IOR_IK_vs._CAST_6DoF	8.44*	11.54*	0.02	0.03
IOR_6DoF_vs._CAST_IK	8.01*	8.64*	0.03	0.03
IOR_6DoF_vs._CAST_6DoF	6.30*	14.01*	0.02	0.01
CAST_IK_vs._CAST_6DoF	4.75	6.29*	0.02	0.03

For kinematic parameters, the most pronounced discrepancies were observed in KFA. Particularly, KFA differences exceeded the clinical threshold of 5° in all comparisons except IOR_6DoF vs. CAST_6DoF in the contralateral side, with peak values up to 17.42° (CAST_IK vs. IOR_6DoF, contralateral side). Similarly, all MAD values for KAA exceeded 5°, with a maximum difference of 11.84° on the contralateral side and 11.43° for the TKA one (IOR_6DoF vs. CAST_IK for both). Regarding KIRA, most protocol comparisons yielded clinically meaningful discrepancies, with all MAD value exceeding the threshold, except for CAST_IK_vs._CAST_6DoF relative to the TKA side. The largest differences were observed between IOR_IK vs. CAST_IK, reaching 15.48° on the contralateral side.

In the context of kinetic parameters, MAD values ranged from 0.07 to 0.17 Nm/kg for KFM, 0.04–0.10 Nm/kg for KAM, and 0.01–0.04 Nm/kg for KIRM. The highest disagreement was consistently observed in comparisons involving different modeling approaches (IK vs. 6DoF) combined with distinct marker sets (IOR vs. CAST), while pairings using the same constraint method yielded comparatively lower MADs.

Additionally, in line with MAV results, MAD values were systematically higher for the contralateral limb, underscoring a recurrent side-dependent pattern.

To complement these findings with a measure of effect size, Cohen’s *d* with 95% confidence intervals were computed for all pairwise comparisons between protocol configurations and biomechanical parameters. These values, reported in Supplementary Table S1, provide an additional quantitative estimate of the magnitude and consistency of protocol-induced differences across biomechanical variables.

### 3.3 Time-resolved differences: SPM analysis

**FIGURE 2:**
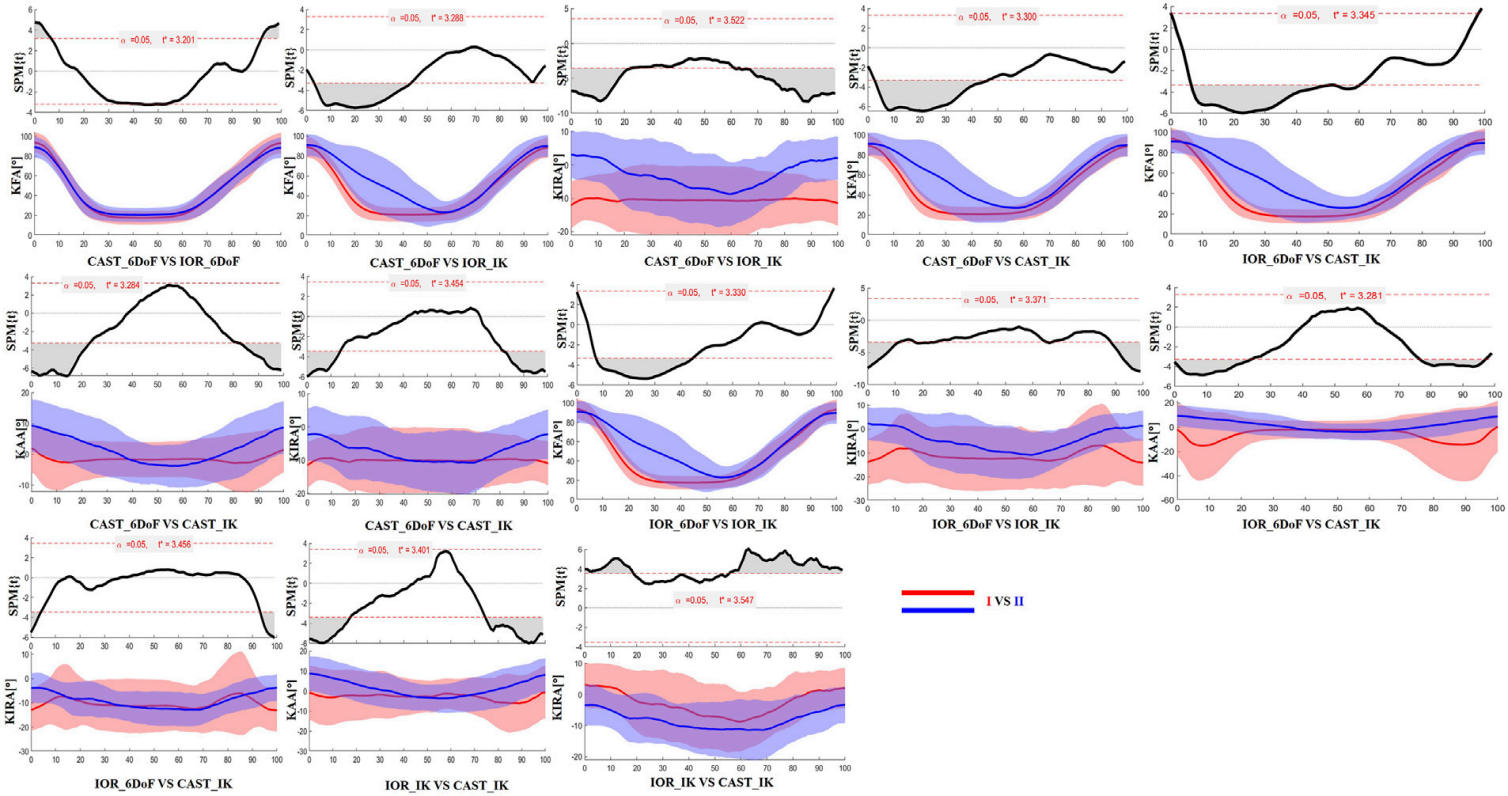
SPM results comparing protocol-dependent differences in knee joint angles over the time-normalized STS task for the limb scheduled for TKA. Each subplot shows the SPM statistic (top) and mean ± SD waveforms (bottom) for two protocol configurations (I VS II). Statistically significant time intervals are shaded in grey in the SPM plots. Red and blue curves indicate the two protocols being compared, and the critical threshold (t*) is indicated by the dashed red line in each plot.

**FIGURE 3:**
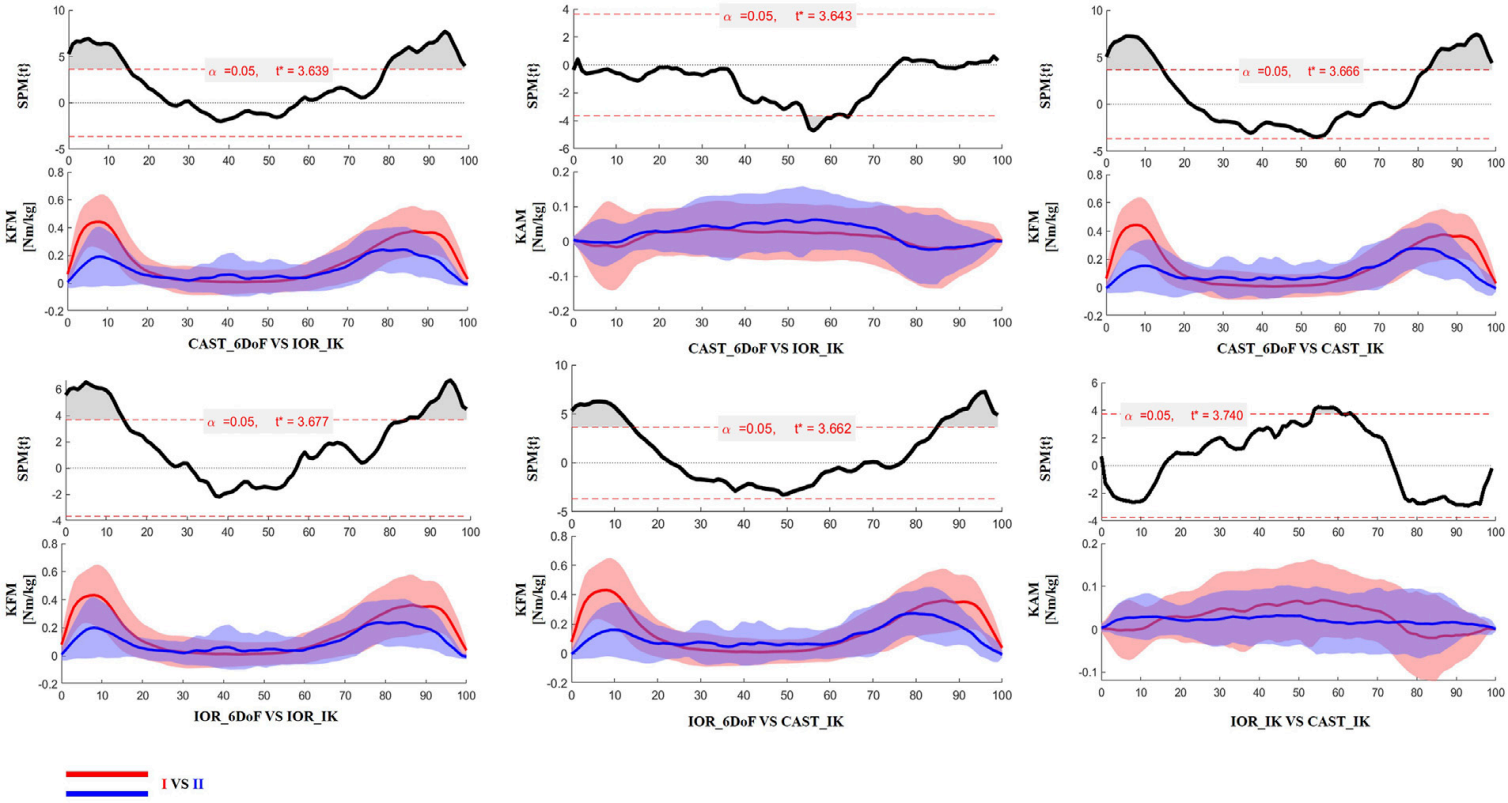
SPM results comparing protocol-dependent differences in knee joint moments over the time-normalized STS task for the limb scheduled for TKA. Each subplot shows the SPM statistic (top) and mean ± SD waveforms (bottom) for two protocol configurations (I VS II). Statistically significant time intervals are shaded in grey in the SPM plots. Red and blue curves indicate the two protocols being compared, and the critical threshold (t*) is indicated by the dashed red line in each plot.

**FIGURE 4:**
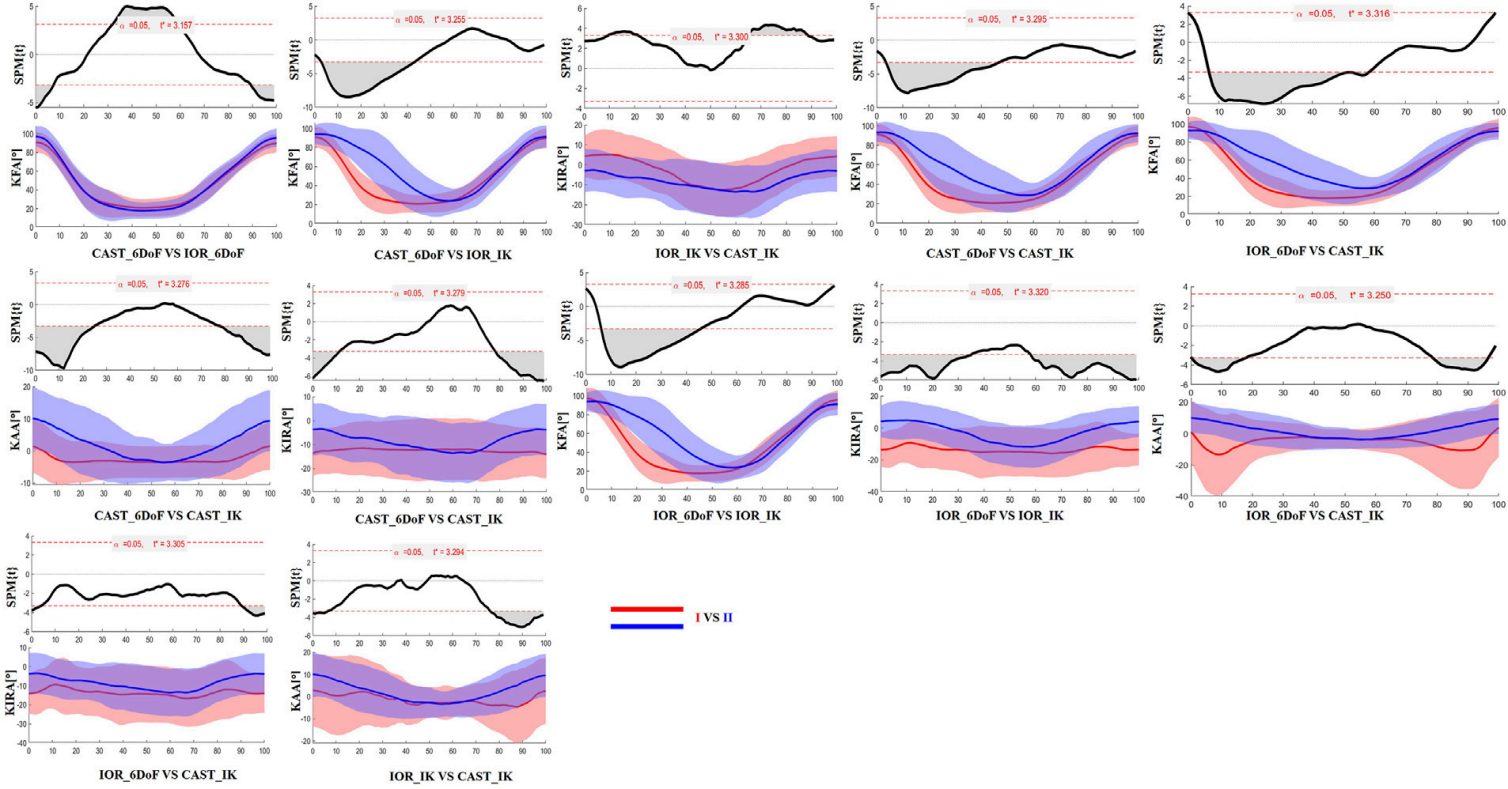
SPM results comparing protocol-dependent differences in knee joint angles over the time-normalized STS task for the contralateral limb. Each subplot shows the SPM statistic (top) and mean ± SD waveforms (bottom) for two protocol configurations (I VS II). Statistically significant time intervals are shaded in grey in the SPM plots. Red and blue curves indicate the two protocols being compared, and the critical threshold (t*) is indicated by the dashed red line in each plot.

**FIGURE 5:**
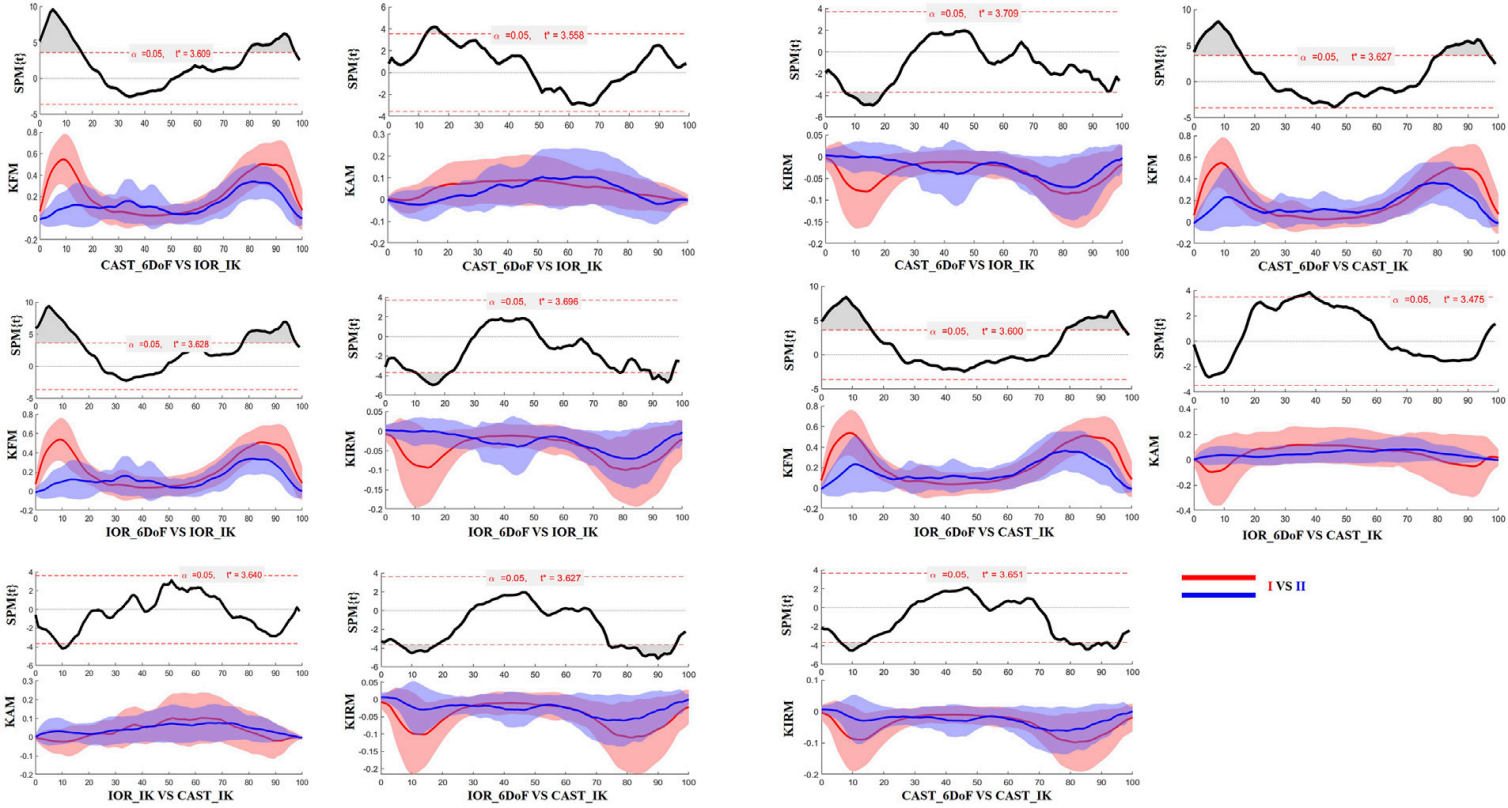
SPM results comparing protocol-dependent differences in knee joint moments over the time-normalized STS task for the contralateral limb. Each subplot shows the SPM statistic (top) and mean ± SD waveforms (bottom) for two protocol configurations (I VS II). Statistically significant time intervals are shaded in grey in the SPM plots. Red and blue curves indicate the two protocols being compared, and the critical threshold (t*) is indicated by the dashed red line in each plot. Knee joint moments are expressed as Nm/kg.

The SPM results are presented in Figures 2, 3 for the limb scheduled for TKA, and in Figures 4, 5 for the contralateral limb. Each figure displays the SPM statistic and corresponding mean curves for all protocol combinations in both kinematic and kinetic parameters. Statistically significant differences are highlighted in grey. Across both limbs, significant differences were observed in multiple parameters, and these discrepancies were often confined to the transition from sitting to standing or the final stabilization phase, underscoring the sensitivity of protocol combinations to dynamic joint behavior.

**FIGURE 6:**
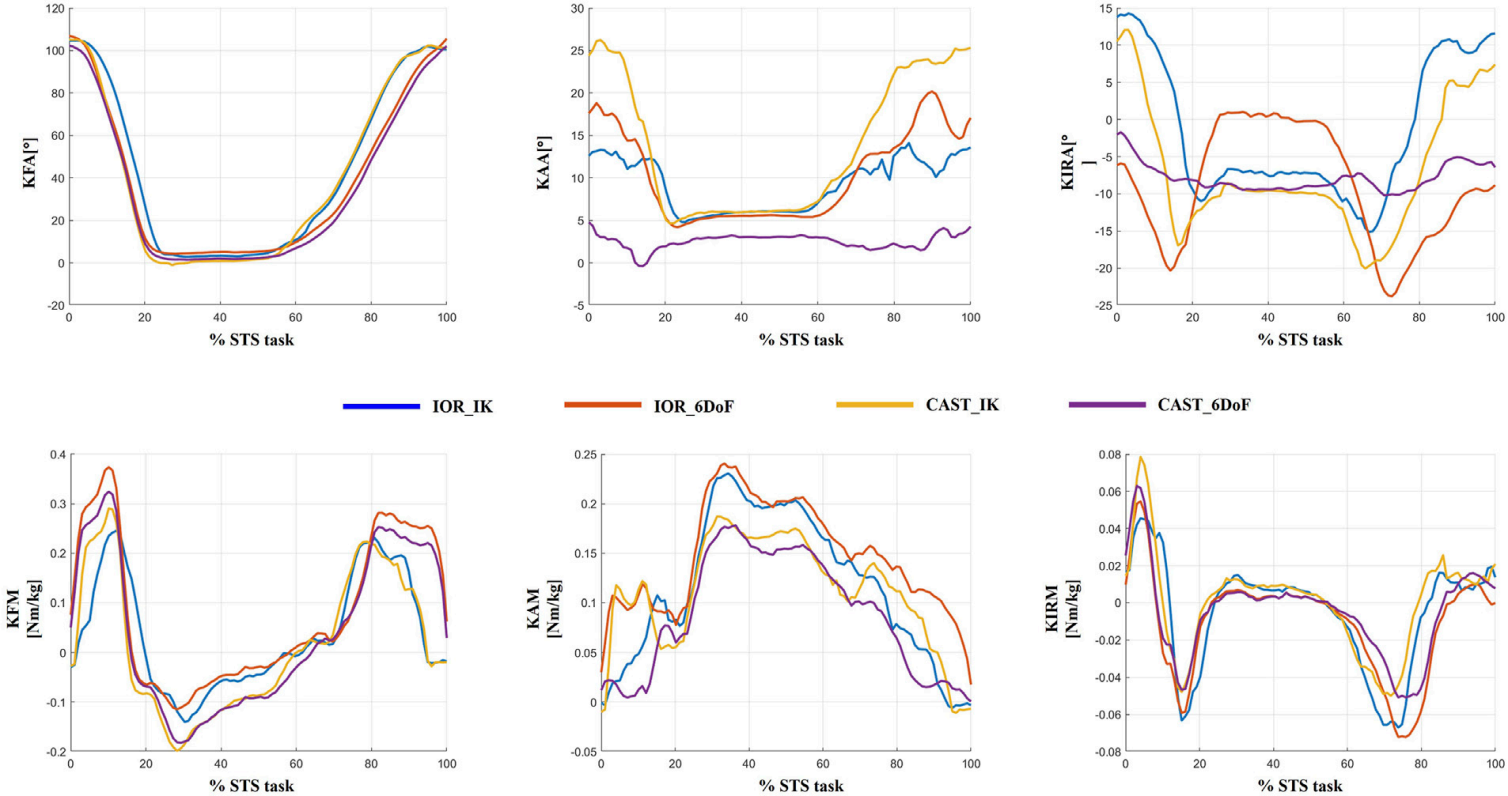
Example of inter-protocol variability in a single representative patient. Joint angles and joint moments obtained with the four protocol configurations (IOR-IK, IOR-6DoF, CAST-IK, CAST-6DoF) are shown over the full time-normalized sit-to-stand cycle. Differences in magnitude and temporal features across waveforms emphasize the influence of protocol selection on individual-level biomechanical interpretation.

To visually exemplify the protocol-induced variability observed at the individual level, Figure 6 displays joint kinematics and kinetics from a single representative patient, processed using all four protocol configurations (IOR-IK, IOR-6DoF, CAST-IK, CAST-6DoF). Variations are particularly evident in frontal and transverse planes, reflecting the combined effects of segment definition, joint constraints, and tracking strategy. Specifically, the adduction and internal rotation angles showed marked differences in both magnitude and waveform morphology. These discrepancies, if unrecognized, could lead to inconsistent clinical interpretation of joint biomechanics, particularly in parameters commonly used for assessing disease severity or surgical indication. This qualitative example reinforces the findings from the MAV, MAD, and SPM analyses, highlighting the importance of methodological consistency in biomechanical assessment and interpretation.

## 4 Discussion

The STS task is one of the most biomechanically demanding and functionally relevant movements in daily life (Sonoo et al., 2019; Petrella et al., 2021; O’Keeffe et al., 2023), particularly in patients with KOA (Spyropoulos et al., 2013; Tarnita et al., 2020; O’Keeffe et al., 2023; Hughes et al., 1994; Higgs et al., 2019; Miura et al., 2018). Due to the high loads involved and the critical neuromuscular coordination required, STS assessment provides unique insights into lower limb function, compensatory strategies, and disease progression (Spyropoulos et al., 2013; Patsika et al., 2011; Petrella et al., 2021; Jeon et al., 2019). In this context, obtaining accurate and consistent measures of joint kinematics and kinetics is essential. However, despite the increasing use of OMC in the field of KOA research (Spyropoulos et al., 2013; Pan et al., 2023; Petrella et al., 2021; Anan et al., 2015; Soloklo et al., 2025; Pan et al., 2024), the impact of protocol choice during this functional task remains poorly investigated.

To fill this gap, the present study aimed to systematically evaluate how different marker sets (IOR and CAST) and biomechanical models (IK and 6DoF) affect the estimation of knee joint angles and moments during the STS in patients with end-stage KOA.

Previous studies have widely documented how methodological choices in motion analysis can significantly influence the estimation of knee joint biomechanics during gait (Buczek et al., 2010; Collins et al., 2009; Duffell L. et al., 2014; Ferrari et al., 2008; Langley et al., 2021; Mantovani and Lamontagne, 2017; Mentiplay and Clark, 2018; Żuk and Pezowicz, 2015). Importantly, while gait studies have reported high consistency in sagittal plane biomechanics (Ferrari et al., 2008; Langley et al., 2021; Mantovani and Lamontagne, 2017; Mentiplay and Clark, 2018; Baudet et al., 2014; Duffell L. D. et al., 2014), our results regarding STS revealed substantially greater variability. For example, Ferrari et al. (2008) reported a MAV of 3.5° for KFA and 0.09 Nm/kg for KFM while our analysis revealed markedly higher values: 21.15° and 23.99° for KFA, and 0.17 Nm/kg and 0.24 Nm/kg for KFM (TKA and contralateral sides, respectively). Similarly, previously reported MADs for KFA during gait have been as low as 0.2° (Mentiplay and Clark, 2018), whereas our data revealed MADs up to 16.01° (TKA side) and 17.42°(contralateral side) for the same parameter. However, it is important to note that the most substantial differences emerged when both the marker set and the modeling constraints differed across protocols. For example, the highest MAD for KFA (17.42°) was observed in the comparison between IOR_IK and CAST_6DoF, combining both a change in anatomical reference and joint DoF. Conversely, using the same model constraints, such as IOR_IK vs. CAST_IK, and IOR_6DoF vs. CAST_6DoF, showed lower MADs for KFA (8.76° and 4.15°, respectively). Similar patterns were observed for KFM where the pair “IOR_6DoF vs. CAST_6DoF” showed the lowest values (0.05 Nm/kg for both limbs). These results suggest that the interaction between marker placement strategy and joint modeling assumptions has a compounding effect on biomechanical outputs, which becomes particularly evident in high-demand and large angular excursions tasks such as those occurring in the sagittal plane during STS.

With respect to non-sagittal planes, prior gait studies have consistently highlighted the elevated sensitivity of frontal and transverse joint biomechanics, key indicators of disease severity and progression (Aljehani et al., 2022; Duffell L. D. et al., 2014; Duffell et al., 2017; Rivière et al., 2017; Clément et al., 2019; Gu et al., 2022; Okamoto et al., 2024), to differences in motion analysis protocols (Ferrari et al., 2008; Langley et al., 2021; Mantovani and Lamontagne, 2017; Mentiplay and Clark, 2018; Baudet et al., 2014; Duffell L. D. et al., 2014). Our results corroborate and expand these observations in the context of the STS task. For instance, all protocol combinations exceeded the clinical threshold for KAA, and most did so for KIRA, with maximum discrepancies approaching 12°. Interestingly, the lowest inter-protocol disagreement in these planes was observed between the two CAST-based models, suggesting that cluster configurations may help reduce measurement inconsistencies. This is biomechanically consistent, as such setups are specifically designed to attenuate soft tissue artifacts (Buchman-Pearle and Acker, 2021; Duffell L. et al., 2014; Duffell L. D. et al., 2014; Ji et al., 2023), which disproportionately affect rotational measures outside the sagittal planes (Ferrari et al., 2008; Langley et al., 2021; Mantovani and Lamontagne, 2017; Mentiplay and Clark, 2018; Camomilla et al., 2017).

These results should be interpreted considering that most gait-related validation studies were conducted on healthy populations and often involved smaller sample sizes than the present study (Mentiplay and Clark, 2018; Ferrari et al., 2010; Langley et al., 2021; Duffell L. et al., 2014; Mantovani and Lamontagne, 2017). The inclusion of patients with end-stage KOA in our cohort adds further clinical relevance, as the presence of joint deformities, asymmetries, and altered motor control may accentuate the variability induced by different protocols.

An additional finding of this study concerns the consistent differences observed between the limb scheduled for TKA and the contralateral side. Both MAV and MAD analyses revealed systematically higher variability in the contralateral limb across all parameters. This suggests that the so-called “less affected” side, may present greater inconsistencies in biomechanical tracking. This evidence is further supported by the SPM results, which showed statistically significant time-localized differences in joint angles and moments across protocol comparisons, often more pronounced in the contralateral limb. Although seemingly counterintuitive, this result may reflect the presence of patient-specific compensatory mechanisms or asymmetric loading strategies involving the contralateral limb. Such adaptations, developed over time to reduce discomfort or protect the symptomatic side, could lead to highly individualized motor patterns (Zeng et al., 2022; Liu et al., 2025) that, in turn, increase inter-protocol variability.

Finally, the results of SPM analysis provided further insight into the time-specific nature of protocol-induced variability. Across both limbs, significant differences between protocol configurations were predominantly observed at the beginning and end of the STS task. These phases are characterized by rapid angular accelerations and lower inter-subject consistency, making them particularly vulnerable to differences in model assumptions and marker tracking sensitivity (Camomilla et al., 2017). It is plausible that subtle shifts introduced by protocol-specific segmentation criteria, particularly in the detection of movement onset and end, may have contributed to the observed temporal misalignments and biomechanical waveform discrepancies.

### 4.1 Limits and future perspectives

This study provides novel insights into protocol-induced variability during STS, but some limitations must be acknowledged. First, the absence of a reference measurement, such as biplanar fluoroscopy or dynamic magnetic resonance imaging (Tersi et al., 2013; Westphal et al., 2013), prevents definitive conclusions about the absolute accuracy of each protocol configuration. Our analysis was designed to address relative variability, rather than quantify the absolute accuracy of each protocol. Second, although the segmentation of the STS task was carefully implemented, the lack of universally accepted event definitions may have contributed to subtle temporal discrepancies, especially at task onset and termination. Third, no correction for multiple pairwise comparisons was applied in the SPM analysis. While this may increase the risk of type I error across protocol combinations, the use of Random Field Theory helps mitigate false positives within each individual test. Fourth, the generalizability of the findings is limited by the standardized experimental setup, including a fixed chair height and the use of arm assistance. Although upper limb use was intentionally allowed and standardized across all participants, it may have influenced kinematic and kinetic outcomes by reducing lower-limb demand and potentially introducing a source of bias. Fifth, even if care was taken in marker placement, the potential influence of soft tissue artefacts, particularly in anatomical marker-based models, should be acknowledged. Finally, our study was limited to a single functional task, while highly informative, additional exercise as stair ascent/descent, or sit-to-walk transitions could provide further insight into task-specific protocol sensitivity.

Future research should focus on task-specific standardization of motion analysis protocols, particularly in high-demand functional movements. In addition, future studies should systematically investigate side-specific factors such as limb dominance and asymmetrical disease severity, which may contribute to the enhanced variability observed in the contralateral limb. Moreover, while this study focused on patients with end-stage KOA, future investigations should also consider other musculoskeletal or neurological conditions where altered motor control and joint loading patterns may further amplify protocol-dependent variability.

### 4.2 Conclusion

This study systematically evaluated the impact of marker set configuration and joint modeling approach on knee biomechanics during the STS in patients with end-stage KOA. Our findings revealed substantial protocol-induced variability highlighting the need for methodological consistency in both research and clinical gait laboratories. These results emphasize the importance of task-specific standardization to ensure the reliable interpretation of functional biomechanics in pathological populations.

## Data Availability

The datasets and scripts supporting the conclusions of this article can be provided by the corresponding author upon reasonable request.

## Abbreviations

KOA, Knee osteoarthritis; STS, Sit to stand; OMC, Optoelectronic Motion Capture; DoF, Degrees of Freedom; IOR, Istituto Ortopedico Rizzoli; CAST, Calibration Anatomical System Technique; Fs, Sampling frequency.
