# Supplementary material for: Joint modeling and marker set selection significantly influence functional biomechanics in end-stage knee osteoarthritis: evidence from the sit-to-stand task
Source: Front Bioeng Biotechnol. 2025 Oct 13;13:1677244. doi: 10.3389/fbioe.2025.1677244 (PMC12554728; doi:10.3389/fbioe.2025.1677244)
Supplement: Supplementary file 1 [file DataSheet1.pdf]

Supplementary Material

1 Supplementary Figures and Tables

1.1 Supplementary Table

Supplementary Table 1. Cohen's *d* effect sizes with 95% confidence intervals (CI) for all pairwise comparisons between protocol configurations (IOR-IK, IOR-6DoF, CAST-IK, CAST-6DoF) across the analyzed kinematic and kinetic parameters. The table also reports the mean of signed differences and their standard deviation for transparency.

Parameter	Protocol Pair	Mean Difference	Std Difference	Cohen d	CI_lower	CI_upper
KFA	IOR_IK_vs_IOR_6DoF	10,34	8,32	1,24	0,66	1,81
KFA	IOR_IK_vs_CAST_IK	-1,99	4,79	-0,41	-0,85	0,023
KFA	IOR_IK_vs_CAST_6DoF	10,57	8,24	1,28	0,71	1,85
KFA	IOR_6DoF_vs_CAST_IK	-12,19	7,93	-1,53	-2,17	-0,89
KFA	IOR_6DoF_vs_CAST_6DoF	-0,75	4,73	-0,15	-0,58	0,26
KFA	CAST_IK_vs_CAST_6DoF	12,57	7,64	1,64	0,99	2,29
KAA	IOR_IK_vs_IOR_6DoF	4,77	7,01	0,67	0,18	1,17
KAA	IOR_IK_vs_CAST_IK	-3,95	5,32	-0,74	-1,24	-0,24
KAA	IOR_IK_vs_CAST_6DoF	-0,64	6,02	-0,10	-0,54	0,32
KAA	IOR_6DoF_vs_CAST_IK	-8,02	10,14	-0,79	-1,28	-0,29
KAA	IOR_6DoF_vs_CAST_6DoF	-4,76	10,65	-0,44	-0,88	-0,01
KAA	CAST_IK_vs_CAST_6DoF	3,41	3,67	0,92	0,41	1,44
KIRA	IOR_IK_vs_IOR_6DoF	7,12	6,43	1,10	0,51	1,70
KIRA	IOR_IK_vs_CAST_IK	4,91	4,78	1,02	0,43	1,62
KIRA	IOR_IK_vs_CAST_6DoF	7,78	4,49	1,72	0,96	2,49
KIRA	IOR_6DoF_vs_CAST_IK	-1,24	5,77	-0,21	-0,71	0,28
KIRA	IOR_6DoF_vs_CAST_6DoF	1,12	4,65	0,24	-0,26	0,74
KIRA	CAST_IK_vs_CAST_6DoF	2,79	3,17	0,88	0,32	1,43
KFM	IOR_IK_vs_IOR_6DoF	-0,06	0,05	-1,20	-1,76	-0,63

KFM	IOR_IK_vs_CAST_IK	-0,01	0,03	-0,43	-0,87	0,01
KFM	IOR_IK_vs_CAST_6DoF	-0,06	0,05	-1,18	-1,73	-0,63
KFM	IOR_6DoF_vs_CAST_IK	0,04	0,06	0,73	0,24	1,21
KFM	IOR_6DoF_vs_CAST_6DoF	-0,01	0,06	-0,04	-0,46	0,37
KFM	CAST_IK_vs_CAST_6DoF	-0,04	0,04	-0,97	-1,48	-0,45
KAM	IOR_IK_vs_IOR_6DoF	0,02	0,07	0,34	-0,11	0,80
KAM	IOR_IK_vs_CAST_IK	0,01	0,03	0,24	-0,20	0,69
KAM	IOR_IK_vs_CAST_6DoF	0,01	0,04	0,23	-0,20	0,67
KAM	IOR_6DoF_vs_CAST_IK	-0,01	0,09	-0,05	-0,49	0,37
KAM	IOR_6DoF_vs_CAST_6DoF	-0,01	0,08	-0,08	-0,50	0,34
KAM	CAST_IK_vs_CAST_6DoF	0,01	0,04	0,04	-0,38	0,47
KIRM	IOR_IK_vs_IOR_6DoF	0,01	0,02	0,38	-0,09	0,87
KIRM	IOR_IK_vs_CAST_IK	-0,01	0,01	-0,42	-0,93	0,07
KIRM	IOR_IK_vs_CAST_6DoF	-0,01	0,02	-0,04	-0,52	0,43
KIRM	IOR_6DoF_vs_CAST_IK	-0,01	0,02	-0,57	-1,11	-0,03
KIRM	IOR_6DoF_vs_CAST_6DoF	-0,01	0,02	-0,69	-1,25	-0,14
KIRM	CAST_IK_vs_CAST_6DoF	0,01	0,02	0,24	-0,23	0,71
